# Modeling the Effects of Attentional Cueing on Meditators

**DOI:** 10.1007/s12671-015-0464-x

**Published:** 2016-01-09

**Authors:** Marieke K. van Vugt, Paul M. van den Hurk

**Affiliations:** 1 Institute of Artificial Intelligence and Cognitive Engineering, University of Groningen, Nijenborgh 9, 747 AG Groningen, The Netherlands; 2 Department of Computer Science, Radboud University, Nijmegen, The Netherlands

**Keywords:** Meditation, Computational models, Attention, Decision making, Drift diffusion model

## Abstract

Training in meditation has been shown to affect functioning of several attentional subsystems, most prominently conflict monitoring, and to some extent orienting. These previous findings described the effects of cueing and manipulating stimulus congruency on response times and accuracies. However, changes in accuracy and response times can arise from several factors. Computational process models can be used to distinguish different factors underlying changes in accuracy and response times. When decomposed by means of the drift diffusion model, a general process model of decision making that has been widely used, both the congruency and cueing effects, is subserved by a change in decision thresholds. Meditators showed a modest overall increase in their decision threshold, which may reflect an ability to wait longer and collect more information before responding.

## Introduction

Given the emphasis on attention in meditation instructions, which usually start with “pay attention to your breath”, it is not surprising that many of the early scientific studies of meditation focused on attention (Jha et al. 2007; Slagter et al. 2007; Tang et al. 2007). Those articles showed that the attentional blink was reduced in expert meditators relative to novice controls (Slagter et al. 2007), and in a follow-up study, van Vugt and Slagter (2013) showed that this attentional blink reduction was related to the type of meditation that was practiced. Other studies showed after intensive meditation training, attention can be sustained for longer periods of time (MacLean et al. 2010) and becomes less variable (Lutz et al. 2009).

In addition, Jha et al. (2007) showed that while beginning meditators improved in their ability to orient their attention to relevant locations in space after a mindfulness course, more experienced meditators were better at dealing with conflicting information, and after a retreat additionally improved in their ability to orient themselves to information occurring at a specific moment in time. The finding of improved orienting was replicated in a later study by van den Hurk et al. (2010) in a group of meditators from a different tradition. Another study showed that a specific form of meditation—Integrated Body Mind Training—was associated with improvements in conflict monitoring (Tang et al. 2007). Similarly, conflict monitoring was improved for meditation practitioners, when compared to a relaxation control group (Ainsworth et al. 2013).

**Fig. 1:**
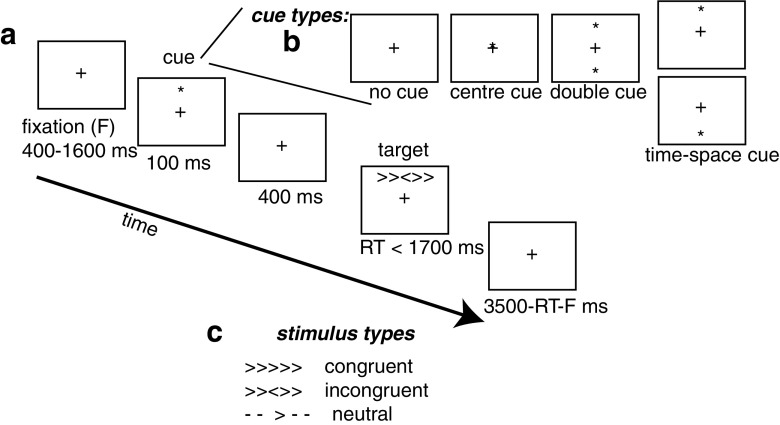
Attentional network task (ANT) paradigm. Participants are asked to respond to the direction of the central arrow. **a** Time line for a trial. **b** Cueing conditions, manipulating the ability to prepare for upcoming information. **c** Congruency conditions, manipulating the ability to deal with conflicting information. Adapted from van den Hurk et al. (2010)

Attentional functioning in many previous studies was assessed with the Attention Network Task (ANT; see Fig. 1), a task in which participants have to judge whether a centrally-presented arrow is pointing left- or right-ward (Fan et al. 2002). This center arrow can point either in the same direction as the flankers (no conflicting stimulus information) or in the opposite direction (conflicting stimulus information). Preparedness is manipulated with cues. A cue before the main stimulus alerts the participant to an upcoming flanker stimulus. Cueing the location on the screen where the upcoming stimulus is likely to be presented gives spatial information. By comparing conditions with different types of cues or information, the orienting, alerting, and conflict monitoring components of attention are assessed.

Computational modeling could help to distinguish whether the response time changes underlying these attentional components are related to changes in speed-accuracy trade-off, bias, or ability to extract information from a stimulus (Voss et al. 2013; Wagenmakers et al. 2007; White et al. 2010). The drift diffusion model (Ratcliff 1978) can help to make this distinction. The drift diffusion model (DDM) is a stochastic differential equation that describes a participant’s responses as arising from a noisy evidence accumulation process. From the start of each trial, evidence drifts towards one of the two decision thresholds, corresponding to the response options (in the ANT “left” and “right”). When the evidence crosses a threshold, the corresponding decision is made. The model’s predicted response time is the time needed to cross the threshold plus some fixed perceptual and motor latencies (“non-decision time”).

The model produces a distribution of response times and accuracies that are compared to a participant’s actual response times and accuracies. Search algorithms are then used to find the model parameters that create the best match between the predicted and observed response times and accuracies. These parameters can be interpreted as the causes of each individual’s behavior. The first parameter is the speed with which the evidence accumulation moves towards a threshold (i.e., the ability to extract information from the stimulus) is the drift rate. The height of the decision threshold captures response caution. A starting point captures decision biases; by starting evidence accumulation closer to the preferred option, a bias is modeled. Fluctuations in sustained attention are captured by variability in the speed of evidence accumulation (“drift variability”); variability in the starting point allows the model to predict fast guesses, while variability in non-decision time allows the model to predict the leading edge of the response time distribution (Smith and Ratcliff 2014). In a previous study (van Vugt and Jha 2011), the parameter that changed after intensive meditation practice was the drift rate, thought to reflect the quality of the evidence on which is decided. An open question is whether that previously reported increase in drift rate generalizes to the ANT, or whether alternatively, the observed effects changes in ANT scores (van den Hurk et al. 2010; Jha et al. 2007) are associated with a different mechanism.

There are several previous applications of the DDM to ANT-like tasks, focusing on the comparison between congruent and incongruent flankers (one of the manipulations in the ANT). Some previous studies showed that incongruent stimuli were associated with a lower drift rate than congruent stimuli (Dillon et al. 2015; Pe et al. 2013; White et al. 2011), but in none of these studies the threshold was allowed to account for congruency effects. In another study where the decision threshold was allowed to vary between conditions (King et al. 2012), this parameter accounted for congruency effects. The authors suggested that this reflected the preparedness for exerting cognitive control, such that a higher decision threshold reflected more cognitive control and reduced impulsiveness.

The aims of this paper are two-fold. First, we asked what model parameters capture the effects of cue type and congruency manipulations, respectively, in the ANT task. Since congruency reflects the ability to handle conflicting information, we hypothesized that the decision threshold (caution in responding) increases for incongruent relative to congruent trials (King et al. 2012). Cues, on the other hand, can help to improve the processing of the information that is presented and therefore could be reflected in increases in the drift rate. Second, we asked what parameters (if any) differed between meditators and controls. Based on our previous experiments, we expected that the drift rate, reflecting the quality of attention, would be higher for meditators.

## Method

### **Participants**

In this paper, we combine two datasets. One of these (Experiment 1) has been reported in a previous publication (van den Hurk et al. 2010). Experiment 1 has a purely cross-sectional design, while experiment 2 has a combined cross-sectional/longitudinal design. In both cases, the experimental and control groups are matched one-by-one on education, age, and gender. For most of the analyses, we use only the first test (pre-intervention) of experiment 2. In experiment 1, 20 meditators (mean age 48.1 years, *SD* = 9.0, range 31–60 years; nine female) and 20 controls (mean age 48.1 years, *SD* = 9.1, range 30–63; nine female) were recruited. Meditators had on average 14.5-year experience (*SD* = 11.1; range 0.25–35 years) and practiced between 60 and 420 min per week. They practiced both concentration meditation (maintaining focus on the meditation object) and insight meditation (primarily training meta-awareness).

For experiment 2, 24 meditators participated in a Vipassana retreat. A retreat is a period of secluded, continuous and intensive group practice of meditation. The retreat group had a mean age of 44.9 years (*SD* = 11.0, range 28–62 years; seven male) and the control group a mean age of 44.8 years (*SD* = 10.9, range 28–63 years; seven male). All subjects were Caucasian. All but one participant in the retreat group had previous meditation experience, which ranged from several months to 27 years.

Across the two experiments, none of the participants had any known psychological or neurological deficits. They all had normal or corrected-to-normal vision. A signed informed consent form was obtained from each participant before the experiment. The study was conducted according to the principles expressed in the Declaration of Helsinki.

### **Procedure**

In experiment 1, the ANT was administered once. In experiment 2, the ANT was administered twice (the day when the retreat began and the day when the retreat finished). In this study, the retreats had a duration varying from 8–11 days. Meditation instructions were similar across retreats, and all retreats were in the Vipassana tradition. As such, mindfulness meditation was practiced, which is composed of both concentration meditation (samatha) and insight (Vipassana) meditation. Whereas during samatha meditation, the practitioner is trained to maintain focus on an object for a (theoretically) unlimited period of time, during Vipassana meditation a specific type of meta-awareness is trained (Teasdale et al. 1995). Retreats were held in meditation centers in the Netherlands and led by teachers with extensive experience in teaching and practicing mindfulness meditation. The control group was tested twice on the ANT with 8–11 days in-between. During this time, the control participants went on with their regular lives.

### **Measures**

Participants were seated in front of a 19-inch computer screen at a distance of 65 cm. Stimuli were presented with Presentation software (Version 10.1, Neurobehavioral Systems, Albany, USA). Participants were asked to respond as quickly and accurately as possible to the direction of a central arrow presented with four flanking stimuli (Fig. 1). The flanking stimuli could be either arrows, pointing in the same or opposite direction of the central arrow or horizontal bars. Participants indicated their responses with mouse-clicks with their left- and right thumbs.

To manipulate the ability to deal with conflicting information, congruency was varied. Matching flankers and central arrows made up the congruent condition, while mismatching flankers and central arrows made up the incongruent condition. The neutral condition consisted of flanking stripes. To manipulate the ability to prepare for upcoming information, cues were used. When one asterisk (“centre cue”) or two asterisks (“double cue”) were presented in the center of the screen preceding the stimulus display, this gave the participant information about the timing of the upcoming stimulus display (which was presented above or below fixation with equal probability). The center cue gives slightly less information than the double cue because it directly replaces the fixation cross making it more difficult to make out. A cue could also be presented at the location of the to-be-presented stimulus display, adding spatial information (“time-space cue”).

The complete task consisted of 24 training trials, followed by three test blocks with 94 trials each. Participants were encouraged to take a break after each block. Each trial started with a variable (400–1600 ms) interval during which a fixation cross was presented. This was followed by a cue (if applicable) presented by 100 ms, followed by the target stimulus after an interval of 400 ms. The target was presented for 1700 ms or until the participant responded. The response-to-stimulus interval was variable subject to the constraint that the trial duration was 3500 ms. All trial types were presented in random order within each block.

### **Data Analysis**

Data were combined between the two experiments, where we only used the pre-retreat data from experiment 2 to make it comparable to experiment 1. We analyzed the data with linear-mixed effects models (Bates et al. 2014). Linear-mixed effect models are able to deal with unbalanced data and are robust to violations of independence. Post hoc *t* tests were performed with R’s lmerTests package (Kuznetsova et al. 2015) with a Sattarthwaite approximation for the degrees of freedom.

The DDM (Ratcliff 1978) was fitted in Matlab by means of the DMA toolbox (VandeKerckhove and Tuerlinckx 2007, 2008). Fitting involves simulating the 5 quantiles (0.1, 0.3, 0.5, 0.7, and 0.9) of the correct and error response time distributions for all conditions. The model parameters that best reproduce these quantiles are found using simplex minimization. Four different models were tried, and the best-fitting model was determined by means of the Bayesian Information Criterion. This best-fitting model was then used to draw conclusions about the involvement of model parameters in attentional cueing.

## Results

We will first show how attentional cueing effects manifest in DDM model parameters. Then, we will examine how that is affected by the practice of meditation. All analyses are done on the combined data of experiment 1 and experiment 2.

### Model Parameters Capturing Stimulus Congruency

Participants performed worse and more slowly on the incongruent (accuracy; *M* = 0.94, *S* *E* = 0.01 and response time; *M* = 661, *S* *E* = 8 ms) relative to the other trials (accuracy; *M* = 0.99, *S* *E* = 0 and response time; *M* = 550, *S* *E* = 4 ms; all post hoc *t* statistics comparing to the incongruent condition > 9.6, *p* < 0.001). These conclusions were substantiated by linear-mixed effects models (main effect of condition on accuracy, *F*(2,162.8)=49.0, *p* < 0.001, and RT, *F*(2,148.4)=589.1, *p* < 0.001).

**Fig. 2:**
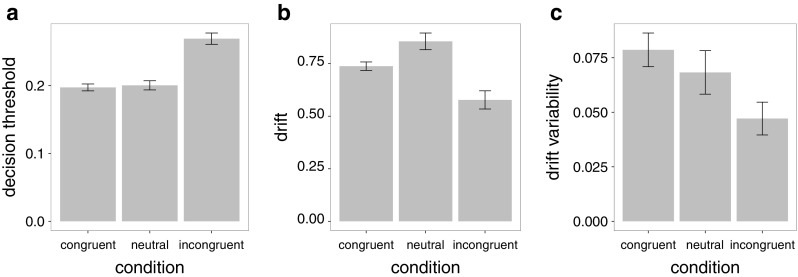
Congruency affects decision thresholds (**a**), drift rates (**b**), and drift variability (**c**). Incongruent stimuli are associated with increased decision thresholds, decreased drift rates, and drift variability. Error bars reflect within-subject standard error of the mean

To investigate which model parameter captures stimulus congruency, we compared a set of models in which different parameters varied across congruency conditions. As shown in Table 1, of the models tried, the best model (i.e., lowest Bayesian Information Criterion, BIC) is one in which the decision threshold, drift rate, and variability in drift rate are all allowed to vary between conditions. There is a main effect of condition on decision threshold (*F*(2,150.5)=33.4, *p* < 0.001; Fig. 2). This parameter is significantly (post hoc *t*>6, *p* < 0.001) larger for the incongruent (*M* = 0.27, *S* *E* = 0.01) than for the other two conditions (*M* = 0.23, *S* *E* = 0.01). The drift rate, reflecting the amount of evidence that can be extracted from the stimulus, also shows a main effect of condition (*F*(2,155.2) = 9.5, *p* < 0.001). It is significantly (post hoc *t*>3.3, *p* < 0.001) lower for the incongruent condition (*M* = 0.58, *S* *E* = 0.04) than for the other two conditions (*M* = 0.79, *S* *E* = 0.04). The same is true for the drift variability, which also shows a main effect of condition (*F*(2158.1)=4.4, *p* < 0.05). Specifically, it is significantly (post hoc *t*>2.0, *p* < 0.05) smaller for the (*M* = 0.05, *S* *E* = 0.01) than for the other conditions (*M* = 0.07, *S* *E* = 0.01). These results demonstrate that stimulus incongruency is associated with a decrease in drift rate, its variability and an increase in response caution relative to congruency.

**Table 1:** Model comparison investigating the effect of stimulus congruency

model	Experiment 1	Experiment 2
	# of participants	# of participants
vary a, *η*, v	23	26
vary a, *η*	9	10
vary a, *η*, v, Ter	0	0
vary a, v, Ter	8	12
total	40	48

The table indicates the number of participants for which each model was the best model with the lowest Bayesian Information Criterion. a = decision threshold; v = drift rate; Ter = non-decision time; *η* = drift variability

### Model Parameters Capturing Cueing Effects

Cues affected behavior in the expected ways (Table 2). There was a main effect of cue type on accuracy (*F*(3,305.0)=12.5, *p* < 0.001). Overall, the more information the cues gave, the more accuracy improved, as all cues were significantly different from the “no cue” condition (post hoc *t* test, all *t*>4.4, *p* < 0.001). Response time also showed a main effect of cue type (*F*(3,304.7)=21.7, *p* < 0.001). Post hoc *t* tests indicated that the no-cue condition led to significantly (all *t*>4.2, *p* < 0.001) slower (*M* = 647, *S* *E* = 7 ms) response times than all other conditions (*M* = 576, *S* *E* = 4 ms). Among the cues, the time-space cue led to significantly (all *t*>2.9, *p* < 0.005) faster response times than the other cues.

**Table 2:** Effects of cues on behavior

	accuracy	(SE)	RT (ms)	(SE)
no cue	0.96	0.005	638	5
center cue	0.98	0.002	583	5
double cue	0.98	0.002	575	5
time-space cue	0.98	0.002	555	5

Accuracy improves and response time becomes faster when cues provide more information

**Fig. 3:**
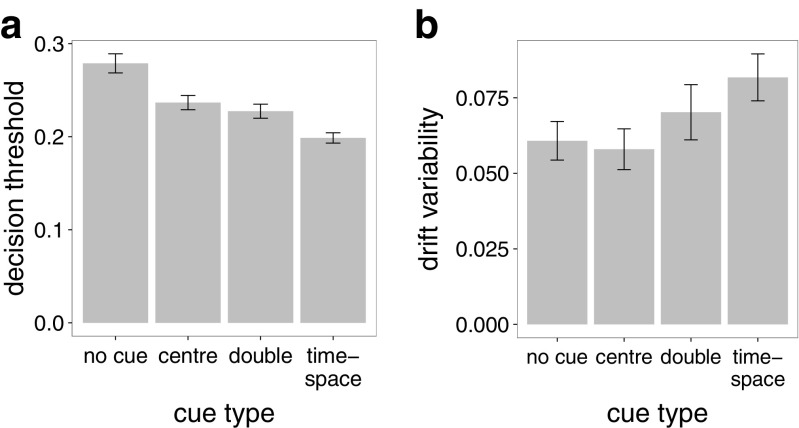
Cues affected decision threshold (**a**) and drift variability (**b**). Decision thresholds decreased and drift variability increased with the amount of information that cues provided. Error bars reflect standard error of the mean

In contrast to congruency, there is no prior literature on what DDM parameters are sensitive to cueing effects. A model comparison revealed that the most successful model is one in which decision threshold, drift, and drift variability vary across conditions (Table 3). All three parameters varied significantly across conditions. Figure 3a shows that the main effect of cue type on decision threshold (*F*(3,141)=69.9, *p* < 0.001) reflects a threshold that decreases with more cue information. The smallest decision threshold occurs for time-space cues (*M* = 0.2, *S* *E* = 0.01), which were different all other conditions (post hoc *t*>3.54, *p* < 0.001). The highest decision threshold was found for the no-cue condition (*M* = 0.28, *S* *E* = 0.01), which was larger than all other conditions (post hoc *t*>6.62, *p* < 0.001). This indicates that participants adjust their decision threshold upon seeing a cue. The time-space cue was also associated with a trend towards a main effect of drift variability (*F*(3,303.6)=2.5, *p* < 0.1). Specifically, drift variability was significantly (post hoc *t*>2.2, *p* < 0.05) larger for the time-space cue (*M* = 0.08, *S* *E* = 0.01) than for the other cue types (*M* = 0.06, *S* *E* = 0; Fig. 3b).

**Table 3:** Model comparison investigating the effect of cueing

model	Experiment 1	Experiment 2
	# of participants	# of participants
vary a, *η*, v	22	25
vary a, *η*	18	15
vary a, *η*, v, Ter	0	0
vary a, v, Ter	0	8

This table lists the number of participants for which each model is the best model (lowest BIC). a = decision threshold; v = drift rate; Ter = non-decision time; *η* = drift variability

### Differences in Model Parameters Between Meditators and Controls

**Fig. 4:**
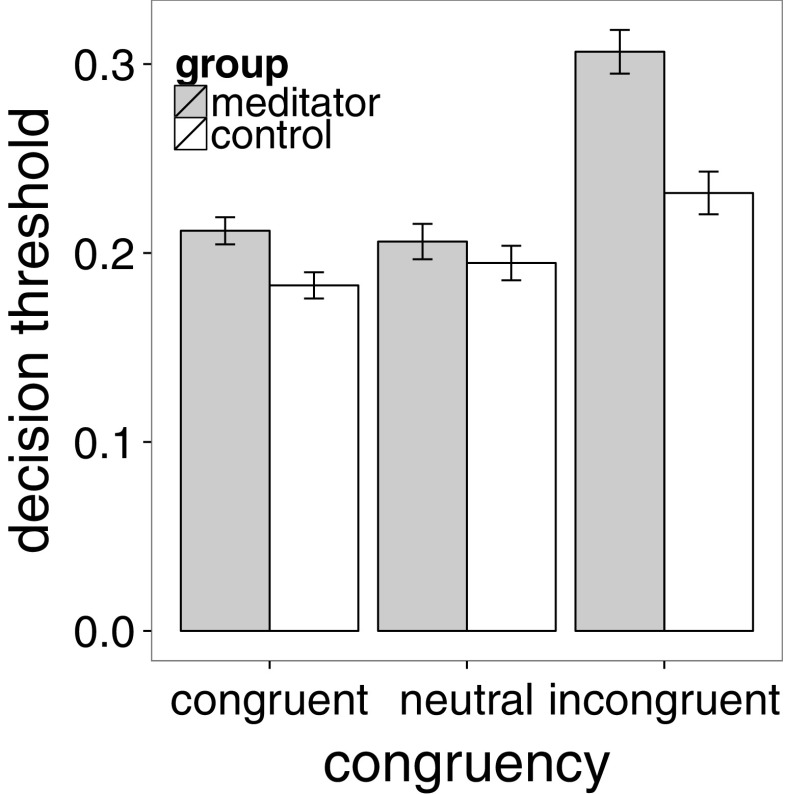
Effects of meditation on the decision threshold parameter. Meditators have a larger decision threshold and adapt it more to the congruency of the stimulus. Error bars reflect standard error of the mean

We then investigated how DDM model parameters differed between meditators and controls (Table 4). There was a significant main effect of group on decision threshold (*F*(1,88.9)=5.8, *p* < 0.05), indicating that the decision threshold was higher for meditators (*M* = 0.25, *S* *E* = 0.01) than for controls (*M* = 0.2, *S* *E* = 0.01). The significant interaction between congruency and group (*F*(2,151.9)=5.0, *p* < 0.01) indicated that this threshold increase was particularly pronounced for the incongruent condition (post hoc *t* tests indicated the incongruent condition was different from all others; *t*>6.3, *p* < 0.001; Fig. 4). In contrast, there was no difference between meditators and controls as a function of cueing. None of the other parameters differed significantly between meditators and controls.

**Table 4:** Statistics (*F* value, *p* value from the linear-mixed effects model) for the effect of meditation on model parameters

parameter	group	condition × group interaction
congruency manipulation		
a	*F*(1,88.9)=5.8, *p* < 0.05	*F*(2,151.9)=5.0, *p* < 0.01
v	*n*.*s*.	*n*.*s*.
*η*	*n*.*s*.	*n*.*s*.
cue manipulation		
a	*F*(1,86.0)=3.2, *p* = 0.08	*n*.*s*.
v	*n*.*s*.	*n*.*s*.
*η*	*n*.*s*.	*n*.*s*.

a is the decision threshold; v is the drift rate; *η* is the drift variability. *n*.*s*. is the not significant

Finally, we repeated the model fitting separately for the tests before and after the retreat (only experiment 2). We did not observe any change in the decision threshold or any of the other parameters as a function of the retreat (all *F* for interaction between group and time < 1.16).

## Discussion

We set out to describe the underlying decision processes in an attentional cueing task and how these differed between meditators and controls. The manipulation of conflicting information was associated with an increase in the decision threshold (reflecting readiness for cognitive control) and a decrease in drift rate (quality of information) and its variability (sustained attention). For cueing, the more information the cues gave, the lower the decision threshold, higher the drift rate, and higher drift variability.

This mapping of task manipulations on DDM parameters partly concurs with previous studies. Some previous studies have suggested that drift rate is the DDM parameter that reflects stimulus congruency (Dillon et al. 2015; Pe et al. 2013; White et al. 2011). However, in those studies, the decision threshold was not allowed to vary with stimulus congruency, and hence, it could not predict stimulus congruency. Our results are more in agreement with King et al. (2012), who allowed their decision threshold parameter to vary with congruency. The increase in decision threshold for incongruent stimuli is thought to reflect preparedness to exert cognitive control. There have been no previous studies of the effect of cue type on DDM parameters. Here, we showed that decision threshold decreased and drift rate increased for more informative cues. This is in agreement with the idea that cues increase the amount of information that a participant can extract from the stimulus.

Meditation practice only has a modest effect on DDM parameters: we observed a higher decision threshold for the meditators relative to the controls. The difference in decision threshold was particularly pronounced for incongruent information. There were no effects of meditation practice on processing cueing information. Short-term meditation retreats did not affect any model parameters.

Our findings extend previous studies of the ANT that showed that meditation improved orienting scores and conflict monitoring scores (Ainsworth et al. 2013; van den Hurk et al. 2010; Jha et al. 2007; Tang et al. 2007). Viewed through the lens of the DDM, our results indicate that participants improved their ability to deal with conflicting information by increasing their level of response caution specifically for the most difficult condition.

How could meditation practice have affected the decision threshold? According to one view, meditation practice consists of four phases: a focus phase, a mind-wandering phase in which one becomes distracted, an awareness phase in which one becomes aware of the distraction, and a shifting phase in which one reorients attention to the object of focus (Hasenkamp et al. 2012). In particular, the skill of awareness with which people observe their own performance while at the same time not becoming too entangled in it is being trained in meditation (Lutz et al. 2008; Vago and Silbersweig 2012; van Vugt 2015). In fact, it has been suggested that meditation training involves the “continuous monitoring and adjustment of one’s attentional focus” (Malinowski 2013). This monitoring capability may allow them to observe their behavior more precisely, adjusting to the level of conflict present in the stimuli, as we observed in the modulation of decision thresholds. It is also in agreement with previous findings that meditation reduces impulsivity (Hendrickson and Rasmussen 2013; Murphy and MacKillop 2012; Peters et al. 2011). The ability to set the decision threshold more precisely may have been further improved by a reduction in the amount of “mental noise” that tends to be present (Lutz et al. 2009; van Vugt and Jha 2011), allowing for better monitoring of the desired level of control.

Decision threshold adaptations have previously been observed in the context of ADHD, anxiety, and aging. Mulder et al. (2010) showed that participants with ADHD had more difficulty in adjusting their decision threshold in response to changes in the required speed-accuracy trade-off. This suggests that meditation—if it helps to finetune decision threshold setting—may be beneficial to this population. White et al. (2010) showed that high-anxiety participants have also been shown to increase their decision threshold after an error, whereas controls did not. Ratcliff et al. (2006) demonstrated that for people who were aging, the responses slowed due to increases in decision thresholds, reflecting response conservativeness, in combination with non-decision time.

In a previous study of meditators, van Vugt and Jha (2011) observed that engaging in a meditation retreat resulted in an increase in drift rate and decrease in decision threshold in a visual recognition memory task. Although this latter finding of a meditation-induced change in drift rate may appear to contradict the current results, it should be noted that DDM parameters may adaptively change in different tasks. In a visual recognition memory task with difficult-to-distinguish stimuli, most room for improvement will lie in the ability to distinguish signals and noise. In contrast, in a flanker task, stimuli are already easy to distinguish, but performance is determined by the ability to optimally trade off speed and accuracy correctly, and this is where there is room for improvement. Previous work (van den Hurk et al. 2010) suggested that meditators were better able to extract information from stimuli because they were more accurate than controls with the same response time. However, that could also be interpreted as the increase in response caution that we observe.

The effects of meditation on DDM parameters were modest. One reason for such small effects is the cross-sectional design. If individual differences are large, then cross-sectional studies are very insensitive. In addition, in cross-sectional designs, any observed effects could be due to pre-existing differences between the groups unrelated to their meditation practice. Yet, it should be mentioned that our dataset was matched—every participant in the meditator group was matched to a specific individual in the control group on age, education, and gender. We did not observe effects of a short meditation retreat on model parameters. Yet, even with relatively modest differences between groups in task performance, there could still be differences in the strategy that participants use to perform the task. For example, participants could prepare more for incoming stimuli, such that cues have less of an effect. These strategy differences could potentially be distinguished by means of neural activity (Formisano and Goebel 2003; O’Doherty et al. 2007). Future studies should employ such neuroscience measures together with longer-duration longitudinal designs to further explore the effects of meditation practice on the attention system.

In short, we have shown that both cueing and congruency manipulations in the ANT map onto the decision threshold, drift, and drift variability parameters of the DDM. More informative cues increase the model’s drift rate and decrease its decision threshold. Incongruent information results in a decreased drift rate and increased decision threshold. We also showed how meditators showed overall higher decision thresholds, especially for incongruent information. This higher decision threshold may reflect an ability to wait longer to be able to exert sufficient cognitive control. By decomposing behavior into cognitively interpretable parameters, this study helps to build a theoretical basis for how meditation affects cognition.
